# Targeting interleukin-17 receptor B enhances gemcitabine sensitivity through downregulation of mucins in pancreatic cancer

**DOI:** 10.1038/s41598-020-73659-z

**Published:** 2020-10-20

**Authors:** Lung-Hung Tsai, Kai-Wen Hsu, Cheng-Ming Chiang, Hsiu-Ju Yang, Yu-Huei Liu, Shun-Fa Yang, Pei-Hua Peng, Wei-Chung Cheng, Heng-Hsiung Wu

**Affiliations:** 1 Research Center for Cancer Biology, China Medical University, No. 91, Hsueh-Shih Road, North District, Taichung, Taiwan; 2 Drug Development Center, China Medical University, Taichung, Taiwan; 3 Institute of New Drug Development, China Medical University, Taichung, Taiwan; 4 Department of Pharmacology, and Department of Biochemistry, Simmons Comprehensive Cancer Center, University of Texas Southwestern Medical Center, 5323 Harry Hines Boulevard, Dallas, TX 75390 USA; 5 Graduate Institute of Integrated Medicine, China Medical University, Taichung, Taiwan; 6 Institute of Medicine, Chung Shan Medical University, Taichung, Taiwan; 7 Cancer Genome Research Center, Chang Gung Memorial Hospital at Linkou, Taoyuan, Taiwan; 8 Graduate Institute of Biomedical Sciences, China Medical University, Taichung, Taiwan

**Keywords:** Cancer therapeutic resistance, Targeted therapies

## Abstract

Pancreatic cancer is the fourth leading cause of death worldwide due to its poorest prognoses with a 7% 5-year survival rate. Eighty percent of pancreatic cancer patients relapse after chemotherapy and develop early metastasis and drug resistance. Resistance to nucleoside analog gemcitabine frequently used in first-line therapy is an urgent issue in pancreatic cancer treatment. Expression of mucin (MUC) glycoproteins has been shown to enhance chemoresistance via increased cell stemness. Here we show interlukine-17 receptor B (IL-17RB) expression is positively correlated with MUC1 and MUC4 expression in pancreatic cancer cells and tumor tissue. Moreover, IL-17RB transcriptionally up-regulates expression of MUC1 and MUC4 to enhance cancer stem-like properties and resistance to gemcitabine. These results suggest IL-17RB can be a potential target for pancreatic cancer therapy. Indeed, treatment with IL-17RB-neutralizing antibody has a synergistic effect in combination with gemcitabine for killing pancreatic cancer cells. Altogether, these findings provide feasible applications for IL-17RB-targeting therapy in pancreatic cancer treatment.

## Introduction

Pancreatic cancer is the fourth leading cause of death worldwide, about 85% of pancreatic cancer patients being diagnosed with adenocarcinoma^1,2^. In Taiwan, the incidence and mortality of pancreatic cancer has rapidly increased from 1999 to 2012, with indication of a further 20% increase in incidence and 10% in mortality by 2027^3^. Up to 80% of pancreatic cancer are un-resectable by its highly malignant and early metastasis^2^. Chemotherapy is the standard treatment of pancreatic cancer. Moreover, pancreatic cancer cells in most patients leads to resistance to chemotherapy^4,5^. In the past few decades, 5-fluorouracil (5-FU) and Gemcitabine are the standard of care for the treatment of advanced pancreatic cancer^6^. However, the survival benefits of 5-FU and Gemcitabine are still limited with median survival durations of 4.41 and 5.65 months, respectively^7^. Therefore, effective targeted therapies are urgently needed.

Interleukin-17 receptor B (IL-17RB) is a cytokine receptor, which is activated by IL17B and IL17E ligands. IL-17RB can promote Th2 reaction in CD4+ T helper cells in response to asthmatics^8^. In our preliminary studies, overexpression of IL-17RB strongly correlated with post-operative metastasis and inversely correlated with progression-free survival in pancreatic cancer patients^9–11^. Activated IL17B/IL-17RB signaling, which increases chemokine expression via the NF-κB and ERK1/2 pathway, promotes cancer cell invasion, macrophage and endothelial cell recruitment to the primary sites, and cancer cell survival at distant organs^10–12^. Importantly, treatment with monoclonal antibody against the native form of IL-17RB delays the malignancy of pancreatic cancer cells expressing IL-17RB and significantly extends animal survival. Taken together, these results suggest that IL17B/IL-17RB signaling not only emerges as an important regulator of pancreatic cancer growth and metastasis, but is a feasible target for pancreatic cancer treatment.

MUC1 and MUC4 are transmembrane mucins, which are overexpressed during pancreatic intraepithelial neoplasia (PanIN) progression^13^. MUC1 and MUC4 both are high-molecular-weight glycoproteins related to poor prognosis in thyroid papillary carcinoma^14^, oral squamous cell carcinoma^15^, and pancreatic ductal adenocarcinoma (PDAC)^16,17^. Rod-like structures formed around tumor cells by mucins enhances tumor progression and blocks chemotherapy drugs targeting the cancer cells^17^.

In this study, we demonstrate that IL-17RB promotes MUC1 and MUC4 expression at transcriptional level. MUC1 and MUC4 induced by IL-17RB upregulate expression of cancer stemness-related genes, such as SOX2, Nanog, Oct-4, and surface CD44 to facilitate sphere formation. Furthermore, it was observed that MUC1 and MUC4 are involved in IL-17RB-mediated resistance to gemcitabine in pancreatic cancer cells. Inhibition of IL-17RB by neutralization antibody D9 suppresses the cancer-stemness activity and enhances gemcitabine sensitivity in pancreatic cancer cells. Consistently, IHC results from tissue array showed expression of IL-17RB is positively correlated with MUC1 and MUC4. These findings demonstrate IL-17RB can induce gemcitabine resistance and stemness activity via upregulation of MUC1 and MUC4. Moreover, targeting IL-17RB is a feasible therapeutic strategy for pancreatic cancer.

## Results

### Expression of MUC1 and MUC4 correlates with that of IL-17RB in pancreatic cancer cell

**Figure 1:**
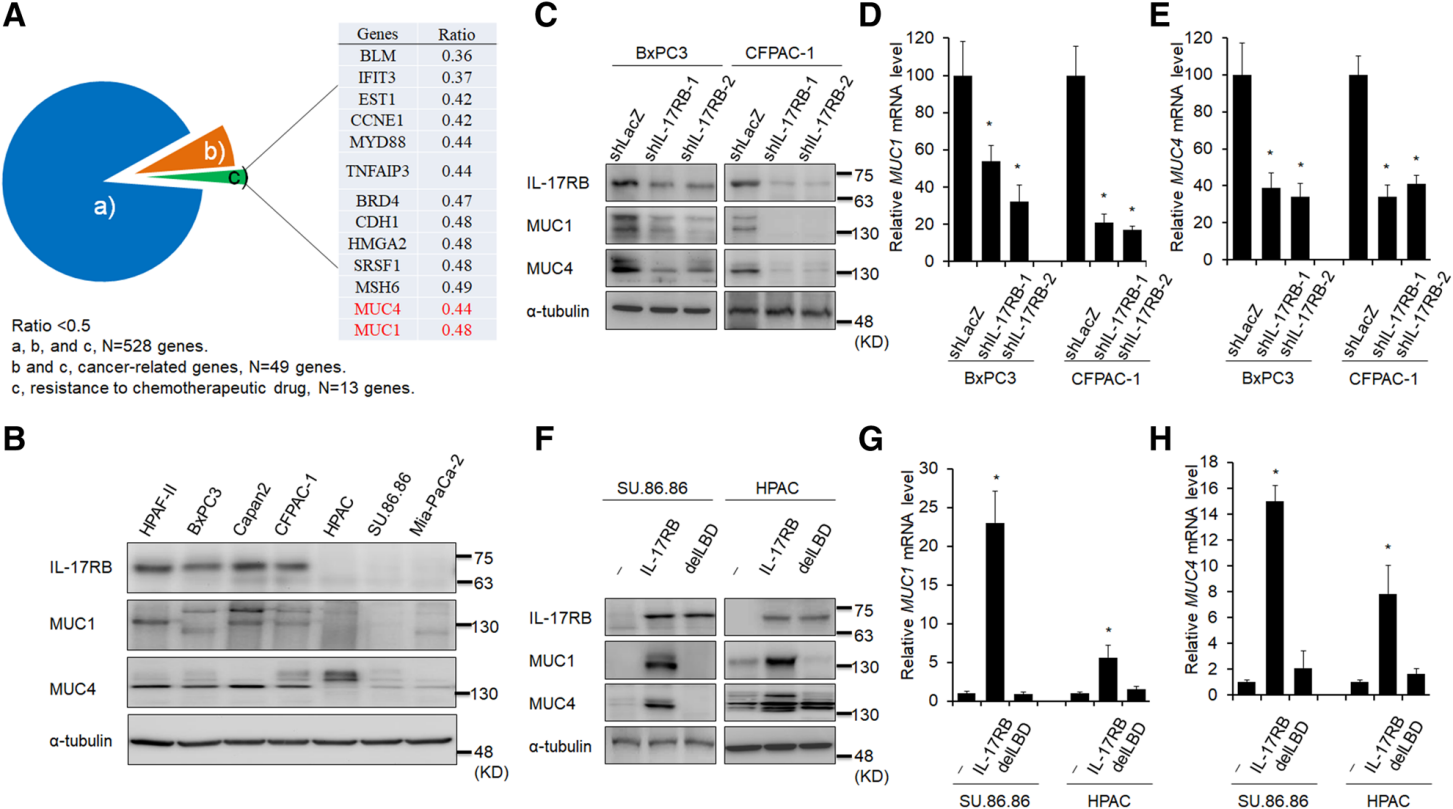
IL-17RB upregulates MUC1 and MUC4 expression in pancreatic cancer cells. (**A**) Pie chart of down-regulated genes (528 genes decreased in more than 50%, a, b and c) analyzed in IL-17RB-knockdown CFPAC1 cells. Cancer-related genes are listed by NCG5.0, n = 49, b and c. Resistance to chemotherapeutic drugs was listed in c. (**B**) Expression of IL-17RB (~ 65KD due to glycosylation), MUC1 (~ 140 KD due to glycosylation), and MUC4 (~ 130 KD due to glycosylation) in pancreatic cancer cell lines was evaluated by immunoblotting with α-tubulin (52 KD) used as a loading control. (**C**–**E**) IL-17RB was knocked down by lentivirus-based shRNA in BxPC3 and CFPAC1 cells. Protein levels of IL-17RB, MUC1, and MUC4 were evaluated by immunoblotting (**C**). mRNA levels of MUC1 (**D**) and MUC4 (**E**) were analyzed by real-time RT-PCR. (**F**–**H**), ectopic wildtype or ΔLBD of IL-17RB was overexpressed in SU.86.86 and HPAC cells. Protein levels of IL-17RB, MUC1, and MUC4 were evaluated by immunoblotting (**F**). mRNA levels of MUC1 (**G**) and MUC4 (**H**) were analyzed by real-time RT-PCR. The asterisk (*) represents a statistical significance with *P* value less than 0.05. The full blotting images were showed in Supplementary Figure 1.

Several membrane receptors associated with pivotal cellular processes are aberrantly overexpressed in cancer cells and have thus emerged as potential targets for receptor-mediated therapeutic strategies. To verify whether IL-17RB upregulates membrane proteins to mediate drug resistance, cDNA microarray data from IL-17RB-knockdown pancreatic cancer cells was used^11^. The oncogenes downregulated by IL-17RB knockdown were selected and validated by immunoblot and qPCR. The results showed 528 genes were downregulated (greater than an 0.5-fold change) by IL-17RB knockdown when compared to the control cells. There are 49 cancer-related genes (from NCG 5.0 analysis identified), including 13 implicated in drug resistance. Only two surface-associated proteins, MUC1 and MUC4, were found in this drug-resistant group (Fig. 1A). To evaluate the correlation between IL-17RB, MUC1 and MUC4, we examined the expression of these genes in a panel of human pancreatic cancer cell lines (Fig. 1B). As expected, the IL-17RB-high-expressed cells, HPAF-II, BxPC3, Capan2, and CFPAC-1, were predicted to have MUC1 and MUC4 expression. In contrast, cells with low expression of IL-17RB, such as HPAC, SU.86.86 and MIA-PaCa-2, showed low MUC1 and MUC4 expression (Fig. 1B). A similar pattern of IL-17RB, MUC1 and MUC4 correlation was also observed at the mRNA level in these cells (Supplementary Fig. 2). These results showed a positive correlation between expression of IL-17RB, MUC1 and MUC4.

To examine whether IL-17RB could regulate MUC1 and MUC4 expression, we knocked down IL-17RB by shRNA in BxPC3 and CFPAC-1 cells, which were feasible for IL-17RB shRNA transduction. Both MUC1 and MUC4 protein levels were decreased in IL-17RB-knockdown cells (Fig. 1C). Downregulation of MUC1 and MUC4 mRNA was also observed in BxPC3 and CFPAC-1 cells by quantitative real-time RT-PCR (RT-qPCR, Fig. 1D,E). Ectopic expression of wild-type IL-17RB upregulated MUC1 and MUC4 expression in SU.86.86 and HPAC cells (Fig. 1F–H). In contrast, ectopic expression of IL-17RB lacking a ligand-binding domain (delLBD), had no effect on the expression of MUC1 and MUC4 (Fig. 1F–H). Together, these results indicate IL17B/IL-17RB signaling is potentially involved in transcriptional regulation of MUC1 and MUC4 expression in pancreatic cancer cells.

### IL-17RB enhances stemness via MUC1 and MUC4

**Figure 2:**
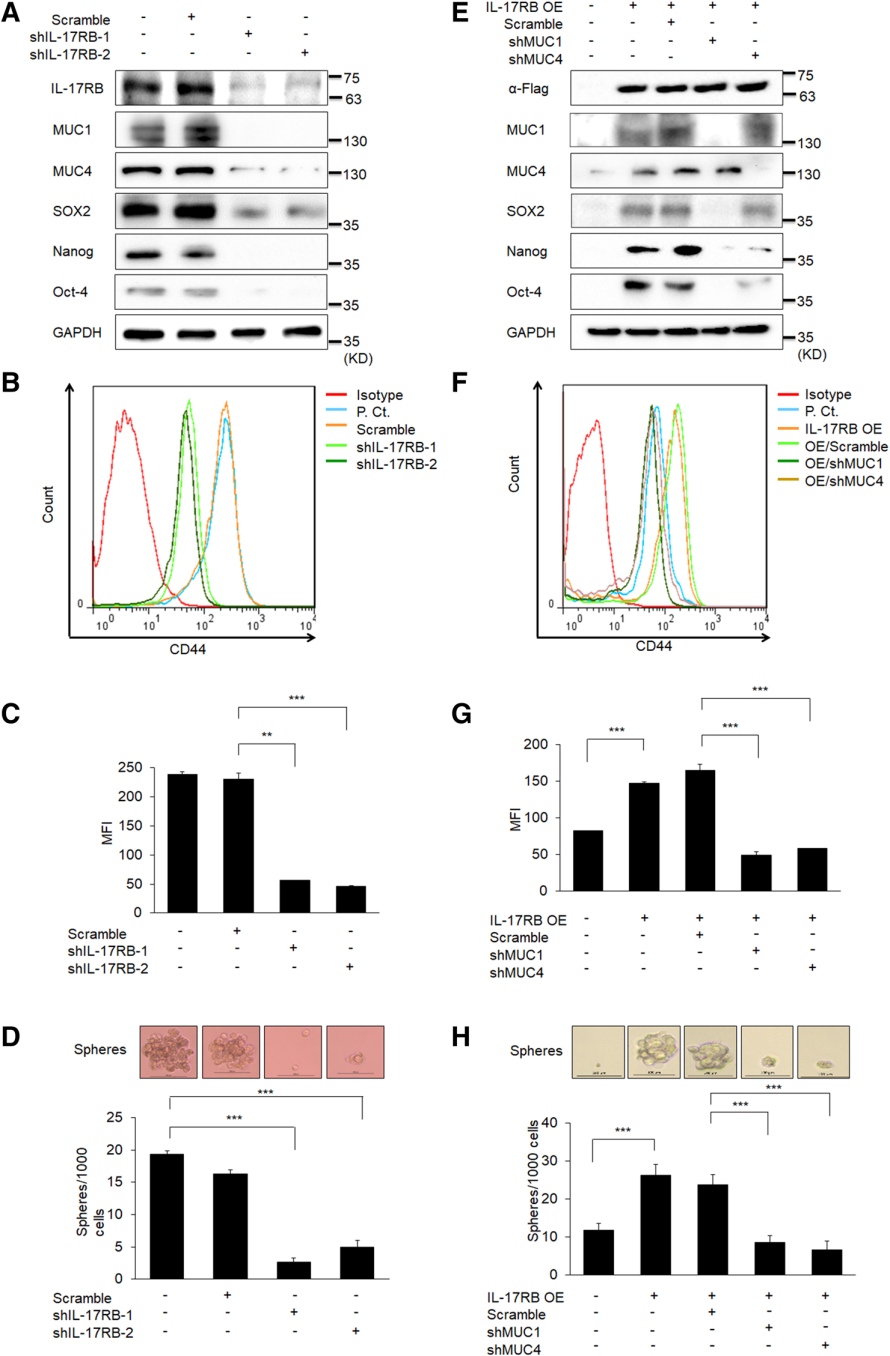
IL-17RB enhances cancer stem cell-like phenotype via upregulation of MUC1 and MUC4. (**A**) Expression of IL-17RB, MUC1, MUC4 and stemness markers SOX2 (40 KD), Nanog (42 KD), Oct-4 (45 KD) in IL-17RB-knockdown BxPC3 cells was measured by immunoblotting. GAPDH (36 KD) was included as a loading control. (**B**, **C**) CD44 expression was measured by flow cytometry, and the MFI (mean fluorescence intensity) was calculated by FlowJo 7.6, and presented in (**C**). (**D**) Sphere formation activity was evaluated by sphere formation assay and the diameter more than 100 µm was calculated. (**E**) Expression of IL-17RB, MUC1, MUC4 and stemness markers SOX2, Nanog, Oct-4 in IL-17RB-overexpressing SU.86.86 cells after transduction with shRNAs of MUC1 and MUC4. GAPDH was served as a loading control. (**F**, **G**) CD44 expression in IL-17RB-overexpressing SU.86.86 cells was measured by flow cytometry, and the MFI (mean fluorescence intensity) was calculated by FlowJo 7.6, and presented in (**G**). (**H**) IL-17RB-overexpressing SU.86.86 cells were transduced with shRNAs of MUC1 and MUC4. Colony formation activity was evaluated by sphere formation assay and the diameter more than 100 µm was calculated. The full blotting images were showed in Supplementary Figure 1.

MUC1 and MUC4 are reported to be involved in stemness which confers drug resistance in cancer cells, implicating that overexpression of IL-17RB may lead to enhancement of chemotherapy resistance through upregulation of these genes in pancreatic cancer cells. MUC1 and MUC4 have been implicated in stem-like features in ovarian cancer^18^. To explore the role of IL-17RB in the cancer stem-like property, we knocked down IL-17RB by lentivirus-based shRNA in BxPC3 cells. Not only MUC1 and MUC4 were suppressed, but the expression of stemness markers, such as SOX2, Nanog and Oct-4, were also decreased in IL-17RB-knockdown cells (Fig. 2A). A decrease of the CD44-positive population was also observed in IL-17RB-knockdown cells (Fig. 2B,C), and downregulation of cancer stemness activity of IL-17RB-knockdown cells was also shown by the sphere formation assay (Fig. 2D). These results indicate endogenous IL-17RB promotes stemness gene expression and stem cell-like sphere formation.

To examine the roles of MUC1 and MUC4 in IL-17RB-mediated stemness in the pancreatic cancer cells, we overexpressed FLAG-tagged IL-17RB, followed by knockdown of MUC1 and MUC4 by lentivirus-based shRNA in SU.86.86 cells. In SU.86.86 cells overexpressing IL-17RB, MUC1 and MUC4 protein levels were increased, and expression of those stemness markers also increased (Fig. 2E). Increased surface CD44 (Fig. 2F,G) and stemness activity were observed (Fig. 2H and Supplementary Fig. 3A). An increase of stemness activity by IL-17RB was also observed in HPAC cells (Supplementary Fig. 3B). Notably, expression of those stemness markers induced by IL-17RB were suppressed by knockdown of MUC1 and MUC4 (Fig. 2E). CD44 expression induced by IL-17RB overexpression was significantly suppressed by MUC1 and MUC4 knockdown (Fig. 2F,G), implicating a critical role of MUC1 and MUC4 in IL-17RB-mediated stemness control. The sphere size and number were also suppressed by MUC1 and MUC4 knockdown in IL-17RB-overexpressing cells (Fig. 2H). Furthermore, sphere formation activity was not increased by delLBD-IL-17RB transfection in SU.86.86 and HPAC cells (Supplementary Fig. 3A,B). It suggested that IL-17RB promotes stemness in ligand-dependent mechanism. Taken together, these results demonstrate IL-17RB enhances stemness-associated gene expression and sphere formation via MUC1 and MUC4 regulation.

### IL-17RB confers gemcitabine sensitivity through MUC1 and MUC4 expression

**Figure 3:**
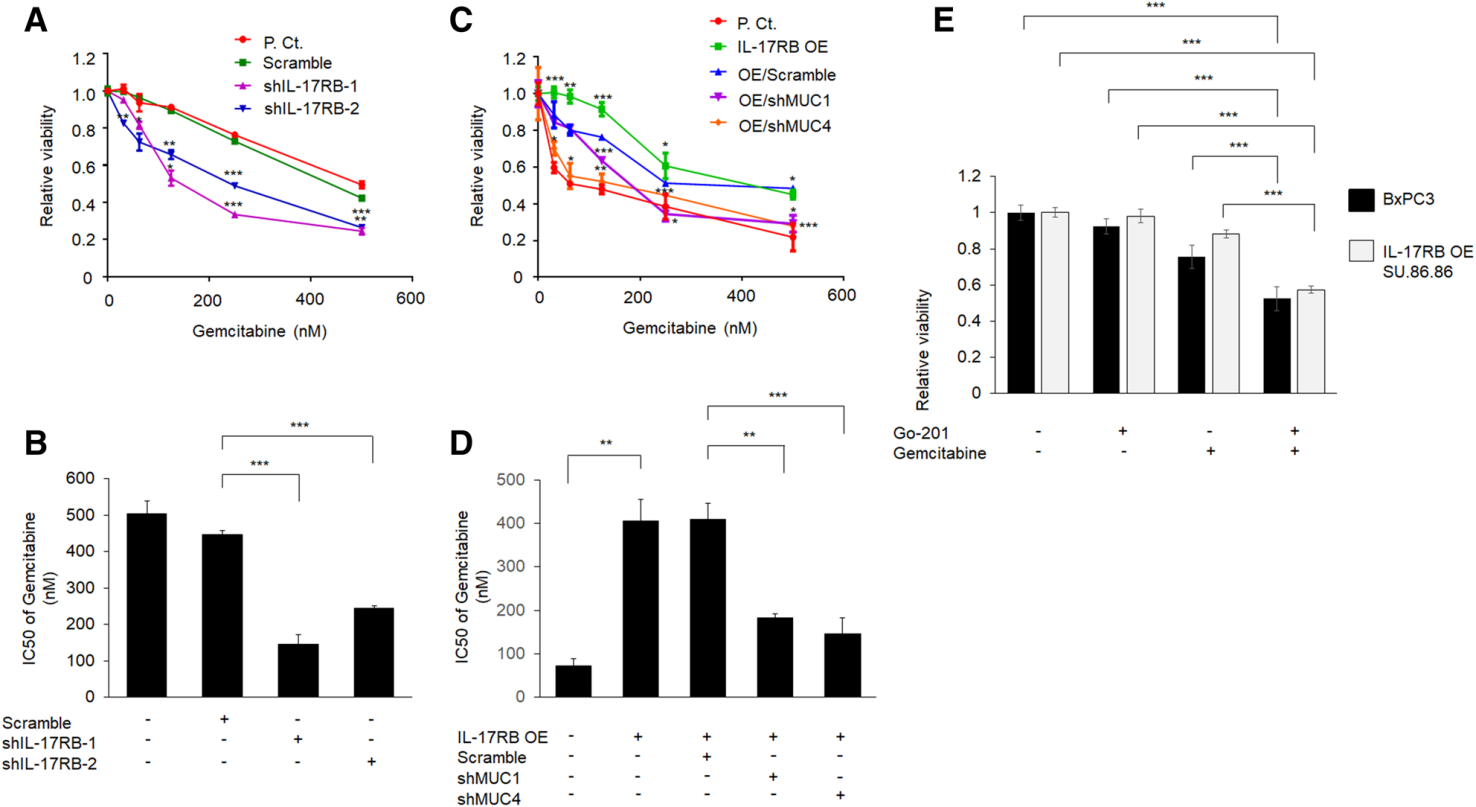
IL-17RB enhances gemcitabine resistance via MUC1 and MUC4. (**A**) cell viability was measured by MTT assay in parental, scramble, and IL-17RB-knockdown BxPC3 cells treated with gemcitabine for 48 h. Statistical significance was calculated by comparison of shIL-17RB-1 or shIL-17RB-2 compared to Scramble. (**B**) The IC50 of gemcitabine toxicity was shown in mean ± SD. (**C**) Cell viability was measured by MTT assay in ectopic IL-17RB-overexpressed, MUC1-knockdown, MUC4-knockdown SU.86.86 cells. Statistical significance was calculated by comparison of IL-17RB OE with P. Ct., and OE/shMUC1 or OE/shMUC4 with OE/Scramble. The IC50 of gemcitabine toxicity was estimated in (**D**). Synergistic effect of MUC1 inhibitor Go-201 (5 μM) and gemcitabine (0.25 µM) on cell viability measured by MTT assay in BxPC3 and ectopic IL-17RB-overexpressing SU.86.86 cells (**E**). Asterisks (*, **, ***) indicated the statistical significance of *P* value less than 0.05, 0.01, or 0.001, respectively.

Overexpression of mucins and enhanced stemness activity were well-known to confer drug resistance in pancreatic cancer treatment^19,20^. To examine the association between IL-17RB and gemcitabine sensitivity in pancreatic cancer cells, the toxicity of gemcitabine was measured by MTT assay in a panel of pancreatic cancer cells. It was observed that pancreatic cancer cells with higher IL-17RB expression (HPAF-II, BxPC3, Capan-2, and CFPAC-1) were more resistant to gemcitabine treatment, and were pancreatic cancer cells with lower IL-17RB level (SU.86.86 and MIA-PaCa2) are more sensitive to gemcitabine treatment (Supplementary Fig. 4). To verify whether IL-17RB could enhance gemcitabine resistance, we measured the toxicity of gemcitabine in IL-17RB-knockdown BxPC3 cells and IL-17RB-overexpressing SU.86.86 cells, respectively. Cell viability after gemcitabine treatment was shown in Fig. 3A–C. The IC50 of gemcitabine was significantly decreased in IL-17RB-knockdown BxPC3 cells (Fig. 3A,B). In contrast, overexpression of IL-17RB could elevate gemcitabine resistance (Fig. 3C,D).

To examine whether MUC1 and MUC4 are involved in IL-17RB-mediated gemcitabine resistance, we knocked down MUC1 and MUC4 in IL-17RB-overexpressing SU.86.86 cells. Cell viability after gemcitabine treatment was shown in Fig. 3C. Overexpression of IL-17RB indeed enhanced gemcitabine resistance in SU.86.86 cells, and knockdown of MUC1 and MUC4 could rescue gemcitabine sensitivity in IL-17RB-overpressing cells. Consistently, treatment of MUC1 inhibitor (Go-201) could suppress IL-17RB-mediated gemcitabine resistance in BxPC3 and IL-17RB-overexpressing SU.86.68 cells (Fig. 3E). Collectively, these results suggest IL-17RB could enhance gemcitabine resistance through upregulation of MUC1 and MUC4 in pancreatic cancer cells.

### Neutralizing antibody of IL-17RB enhances gemcitabine toxicity and suppresses stemness activity

**Figure 4:**
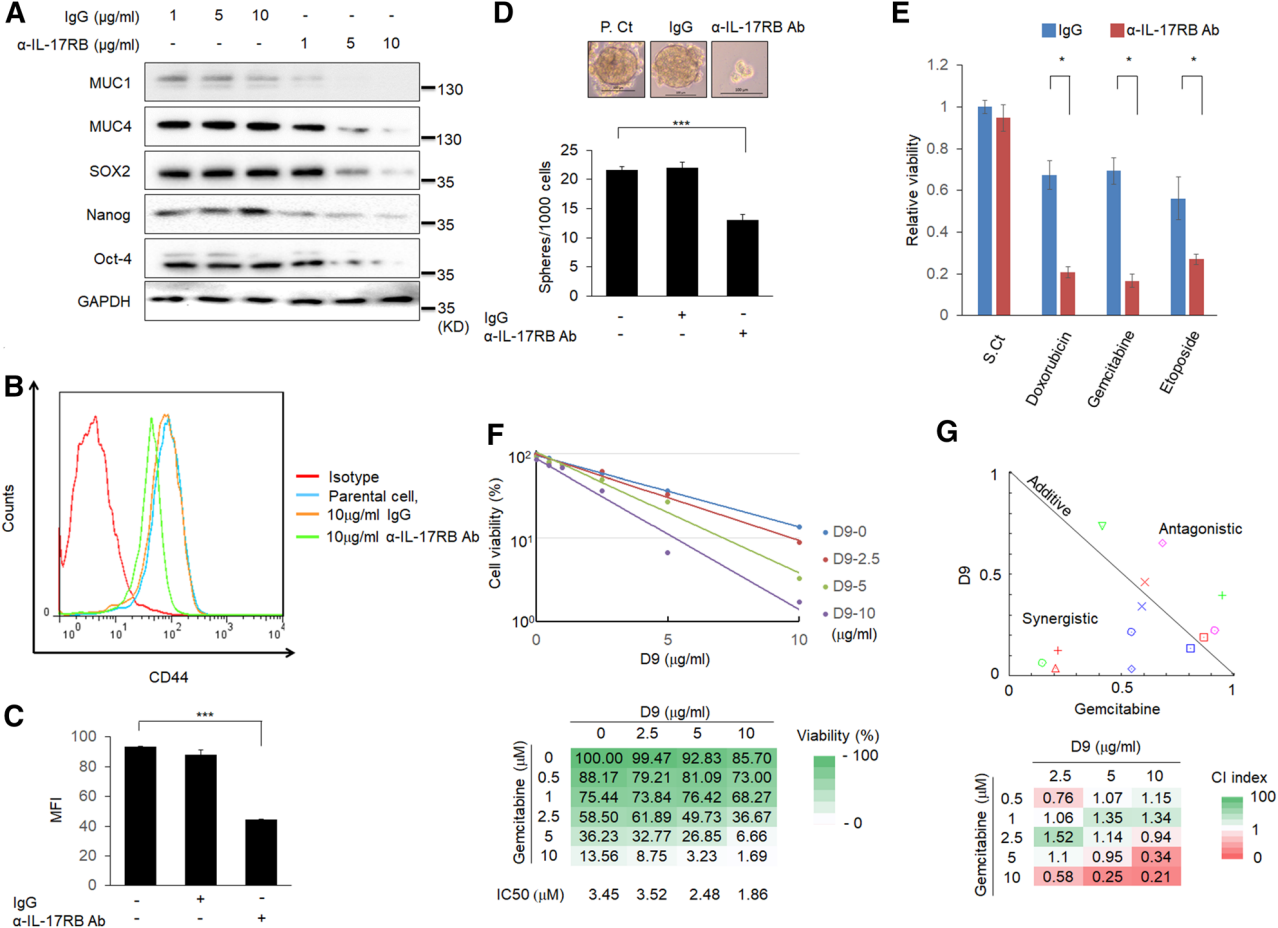
Inhibition of IL-17RB by neutralizing antibody suppresses stemness activity and gemcitabine resistance. (**A**) BxPC3 cells were treated with IgG or neutralizing antibody (D9) with indicated dosages for 48 h and harvested for protein extraction. The expression of MUC1, MUC4, sox2, nanog, and oct-4 was measured by immunoblotting. (**B**) Surface CD44 expression was measured by flow cytometry in BxPC3 with 10 µg/ml IgG or D9 for 48 h. MFI was calculated by FlowJo in (**C**). (**D**) Sphere formation activity was evaluated by sphere formation assay in BxPC3 with 10 µg/ml IgG or D9. (**E**) Cell viability was estimated by MTT following treatment with 0.1 µM doxorubicin, 0.25 µM gemcitabine, or 25 µM etoposide for 48 h in IgG or D9-treated BxPC3. F, cell viability curves were plotted and measured by MTS assay with indicated dosages of D9 and gemcitabine treatment for 24 h in BxPC3 cells. Each cell viability (%) of combined D9 and gemcitabine was shown in the Table. (**G**) normalized isobologram for combination of D9 and gemcitabine treatment in a non-constant ratio was plotted by CompuSyn. The point on the upper-right or lower-left of the line of additivity indicates an antagonistic or synergistic effect, respectively. Combination index (CI) values were calculated by CompuSyn. The full blotting images were showed in Supplementary Figure 1.

Anti-IL-17RB neutralization antibody was reported to be capable of suppressing IL-17RB-mediated pancreatic tumor progression^11^. To examine the therapeutic potential of anti-IL-17RB antibody (D9) to synergize with gemcitabine for pancreatic cancer treatment, the stemness properties of pancreatic cancer cells after anti-IL-17RB (D9) treatment were revealed. Expression of MUC1, MUC4, and stemness associated protein, Sox2, Nanog, and Oct-4, were suppressed after anti-IL-17RB (D9) treatment in BxPC3 cells (Fig. 4A). The surface CD44 was also reduced after D9 treatment (Fig. 4B,C). Furthermore, the size and number of the pancreatic tumor spheres were significantly reduced after anti-IL-17RB (D9) treatment, indicating that targeting-IL-17RB could inhibit cancer stemness activity (Fig. 4D). Therefore, the effect of anti-IL-17RB (D9) combination with chemotherapy was further examined, and it was observed that abrogation of IL-17RB oncogenic signaling by D9 dramatically enhances the cytotoxicity of doxorubicin, gemcitabine, and etoposide in BxPC3 cells (Fig. 4E). To evaluate the combination effect of D9 and gemcitabine on cytotoxicity in pancreatic cancer cells, BxPC3 was treated with combination of D9 (2.5, 5, 10 μg/ml) and gemcitabine (0.5, 1, 2.5, 5, 10 μM) for 24 h. The dose-cytotoxicity effect following D9/gemcitabine treatment was measured by MTS assay and shown in Fig. 4F. D9 treatment dramatically enhanced the cytotoxicity of gemcitabine in a dose-dependent manner. For example, cell viability was reduced from 36.23 to 6.66% with 10 μg/ml D9 treatment and in 5 μM gemcitabine-treated cells also reduced from 13.65 to 1.69% upon 10 μM gemcitabine treatment. Normalized isobologram shows the points with antagonistic or synergistic effects in Fig. 4G. Combination index (CI) (Fig. 4G) was calculated by CompuSyn^21^. The points with synergistic effect were observed under the hypotenuse by D9/gemcitabine combination treatment, especially the points of 5 or 10 μg/ml D9 with 10 μM gemcitabine treatment showed strong synergism (CI < 0.3). These results indicate D9 could reduce gemcitabine usage and be a more effective regimen. Altogether, these results demonstrated a synergistic effect of IL-17RB-targeting therapy with conventional chemotherapy and overcoming gemcitabine resistance.

### IL-17RB expression positively associates with MUC1 and MUC4 expression in pancreatic tumors

**Figure 5:**
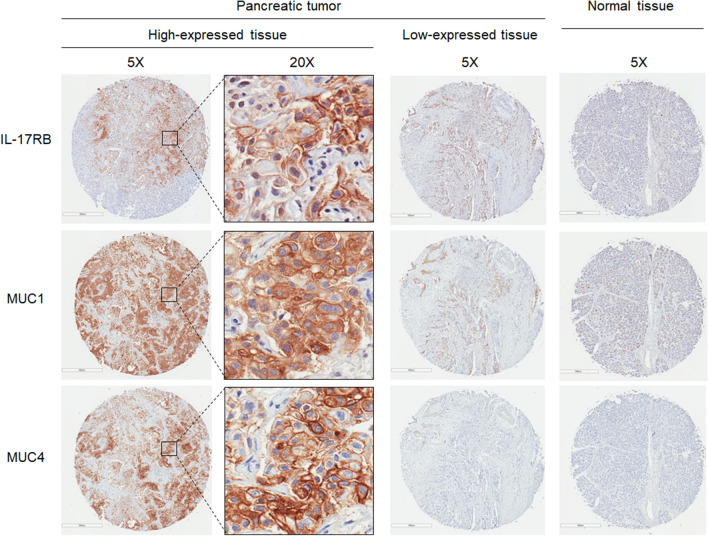
Expression of IL-17RB, MUC1, and MUC4 in pancreatic cancers. Representative IHC staining images of membrane-bound IL-17RB, MUC1, and MUC4 were shown in high expressed tumors at 5× or 20× objective lens, in low expressed tumors at 5× objective lens, and in normal tissues at 5× objective lens.

To investigate the correlation among IL-17RB, MUC1 and MUC4 expression in pancreatic tumors, immunohistochemistry (IHC) was performed to analyze the expression of IL-17RB, MUC1 and MUC4 in 91 pancreatic cancer tumors. The representative results of IL-17RB, MUC1 and MUC4 are shown in Fig. 5. Images of high-expressed, low expressed, and normal tissue were acquired by using a 5× objective lens. Images of the high expressing pattern are enlarged by using a 20× objective lens. IL-17RB, MUC1, and MUC4 are mainly expressed on the surface membrane of pancreatic cancer cells (Fig. 5). Based on the proportion of IL-17RB-expressing cancer cells, the cases can be divided into two groups: Low expression group: negative (0%) and low positive (< 10%); High expression group: high positive (≥ 10%). The clinical parameters including age, gender, tumor subtype, T value, N value, tumor stage and grade are not significantly correlated with IL-17RB, MUC1, or MUC4 expression (Supplementary Table 1). Moreover, the correlation among IL-17RB, MUC1, and MUC4 was presented in Table 1. High expression of IL-17RB strongly correlates with high expression of MUC1 (64%, *P* = 0.020), and high expression of MUC4 (82%, *P* = 0.002).

**Table 1:** Correlation between MUC1, MUC4, and IL-17RB expression in pancreatic cancers.

	n = 91	MUC1 protein		*P* value	MUC4 protein		*P* value
		Low (n = 55)	High (n = 36)		Low (n = 74)	High (n = 17)	
**IL-17RB protein**				0.020			0.002
Low	47	34 (72%)	13 (28%)		44 (94%)	3 (6%)	
High	44	21 (48%)	23 (52%)		30 (68%)	14 (32%)	

The *P* value was tested by Chi-square.

## Discussion

High IL-17RB expression was correlates with poor prognosis of pancreatic cancer, and targeting IL-17RB provides a therapeutic potential for pancreatic cancer treatment^11^. The present work demonstrates that activation of IL-17RB signaling confers pancreatic cancer cells with enhanced cancer stem-like property and resistance to gemcitabine treatment via enhanced MUC1 and MUC4 expression. Lacking the ligand-binding domain of IL-17RB (ΔLBD) had no effect on regulation of MUC1 and MUC4 (Fig. 1G,H). Inhibitors of NF-κB could suppress IL-17RB-mediated MUC1 and MUC4 mRNA expression. Knockdown of IL-17RB, MUC1 and/or MUC4 also suppresses expression of stemness-related markers, such as SOX2, Nanog, Oct-4, and CD44, and inhibited tumor sphere formation. Furthermore, the IHC data show clinical relevance of IL-17RB with MUC1 and MUC4. These findings suggest activation of IL-17RB oncogenic signaling is critical for pancreatic cancer resistance to gemcitabine treatment via MUC1 and MUC4 upregulation.

The underlying mechanisms of gemcitabine resistance in pancreatic cancer cells had been reported in epithelial–mesenchymal transition (EMT)^22^, HMGA1/Akt pathway^23^, and ERK signaling^24^. MUC1 has been reported to be involved in upregulation of MDR genes to facilitate gemcitabine resistance in pancreatic cancer cells^25^. Another mechanism of MUC1 involved in gemcitabine resistance had been reported by stabilization of HIF-1α to increase glucose uptake and pyrimidine biosynthesis^26^. Increase of progenitor cells and gemcitabine resistance by MUC4 overexpression has been observed in pancreatic cancer cells^27,28^. NF-κB pathway is important for MUC4-mediated gemcitabine resistance^28^. In this work, we found IL-17RB upregulates MUC1 and MUC4 through NF-κB pathway (Supplementary Fig. 4) to confer cancer cells resistance to gemcitabine. These findings not only verify the association between IL-17RB overexpression with high expression of MUC1 and MUC4, but also implicate the role of IL-17RB in initiation of oncogenic signaling in pancreatic cancer cells.

Cancer stem cells involved in cancer therapy and drug treatment has been well defined with specific biomarkers^29^. Stemness markers promote cancer stem cell-like formation, such as SOX2, Nanog, Oct-4, CD44, c-Myc, and KLF4, are linked to drug resistance^30–33^. MUC4 regulates CD44 and c-Myc expression via β-catenin in pancreatic cancer cells^34^. In this study, we found stemness activity was elevated by IL-17RB-mediated MUC1 and MUC4, which regulate expression of the stemness formation genes, and lead to gemcitabine resistance (Figs. 2 and 3). Knockdown of MUC1 or MUC4 dramatically enhances stemness via stemness-related genes regulation such as Sox2, Oct4, and Nanog (Fig. 2). But these genes were more significantly down-regulated by knockdown of MUC1 than MUC4, it was suggested other stemness-related genes such as c-Myc and KLF4 might be involved in MUC4-mediated stemness.

Altogether, this study reveals attenuation of the stem-like property by targeting IL-17RB and provide a therapeutic strategy for pancreatic cancer treatment.

## Materials and methods

### Cell lines and inhibitors

Seven human pancreatic cancer cell lines, HPAF-II, BxPC3, Capan2, CFPAC-1, HPAC, SU.86.86, and MIA PaCa-2 cells were obtained from American Type Culture Collection (ATCC). These cell lines are cultured in complete growth medium as the previous study^11^. HPAF-II was maintained in MEM. BxPC3 and SU.86.86 were maintained in RPMI-1640. Capan2 was maintained in MyCoy’s 5A. CFPAC-1 was maintained in IMDM. HPAC was maintained in DMEN/F12. All the media were supplied with 10% FBS. MIA PaCa-2 was maintained in DMEM with 5% horse serum. And the cells were incubated at 37 °C with 5% CO_2_ supplement. All medium supplements were purchased from ThermoFisher (Waltham, MA, US). MEK kinase inhibitor U0126 and PD98059 were purchased from LC Laboratories (Woburn, MA, US)**.** MUC1 inhibitor Go-201 and NF-κB inhibitor BAY11-7082 were purchased from Sigma-Aldrich (St. Louis, MI, US).

### Human pancreatic cancer tissue array

Human pancreatic tissue microarray (PA961e) was purchased from US Biomax, Rockville, MD, US. This tissue microarray contains 78 cases of pancreas adenocarcinoma, one each of carcinoma sarcomatodes, pancreas mixed acinar-neuroendocrine carcinoma, squamous cell carcinoma, neuroendocrine carcinoma and acinic cell carcinoma, three each of pancreas adenosquamous carcinoma and carcinoid, two pancreas solid pseudo-papillary carcinoma, plus five normal pancreatic tissue, single core per case.

### shRNA vectors and transfection

The lentivirus-based shRNA vectors of pLKO.1-shLacZ, IL-17RB (TRCN0000058814, 0000058815), MUC1 (TRCN0000122938), and MUC4 (TRCN0000123299) were purchased from the National RNAi Core Facility (Taipei, Taiwan). Plasmid transfection was performed according to the manufacturer's protocol of Lipofectamine 3000 reagent (ThermoFisher, Waltham, MA, US). Virus particles were packaged in 293 T cells following the manufacturer's protocol of National RNAi Core Facility (Taipei, Taiwan).

### RNA isolation, and real-time reverse transcription PCR assay

Total RNA from cultured cell was isolated using TRIzol reagent (Invitrogen, Waltham, MA, US) and reverse-transcribed with SuperScript IV Reverse Transcriptase (ThermoFisher, Waltham, MA, US) for gene expression analysis according to instructions from the manufacturers. Quantitative real-time RT-PCR was performed using KAPA SYBR FAST qPCR kit (Kapa Biosystems, Wilmington, MA, US) for gene expression according to the manufacturer’s instruction and analyzed on a StepOnePlus Real-Time PCR system (Applied Biosystems, Waltham, MA, US). GAPDH mRNA was used as an internal control for mRNA expression. Expression levels were calculated according to the relative Δ*C*_t_ method. Primer used for detecting on MUC1-forward: **5′-CTCCTTTCTTCCTGCTGCTG-3′**; MUC1 reverse: 5′-*CTGGAGAGTACGCTGCTGGT-3*′*;* MUC4 forward: 5′-CATCACCACCCCCCACAA-3′; MUC4 reverse: 5′-GAAACTCCTCTCTCAGGCAGGAT-3′; GAPDH forward: 5′-GCATTGCCCTCAACGAC-3′; GAPDH reverse: 5′-GTCTCTCTCTTCCTCTTGTGC-3′.

### Gene expression analysis in microarray data

The data of gene expression was analyzed by using Affymetrix U133 Plus 2.0 human oligonucleotide microarrays (Phalanx Biotech Group) in IL-17RB-depleted CFPAC-1 cells as previously^11^. Briefly, a ratio less than 0.5 were selected as candidates while comparison the gene expression in IL-17RB-depleted CFPAC-1 with control. We first obtained 528 genes down-regulated in IL-17RB-depleted cells. To narrow down the range of candidates, we used NCG5.0 (https://ncg.kcl.ac.uk) to identify the cancer-related genes. Out of 13 in 49 cancer-related genes are reported with drug resistance function. And finally the membrane-bound proteins, MUC1 and MUC4, were selected for investigation the mechanism of IL-17RB in drug resistance.

### Immunoblotting

Immunoblotting analysis was performed after electrophoresis using the Gradient Magic SDS-PAGE system (BioEast, Taipei, Taiwan) and transfer to PVDF membrane by Trans-Blot SD Semi-Dry Transfer Cell (Bio-Rad Laboratories, Hercules, Ca, US) at 20 V for 40 min as well as blocking in 5% skim-milk buffer, with overnight incubation of 1:000× dilution of primary antibody, followed by a 1:5000× dilution of horseradish peroxidase-conjugated anti-rabbit or anti-mouse antibody (GeneTex, Hsinchu, Taiwan). Signals were detected by using Clarity Western ECL blotting Substrate (Bio-Rad Laboratories, Hercules, CA, US). The homemade antibody (A81) against IL-17RB was used. Antibodies against MUC1 (VU4H5) and Oct-4 were purchased from Cell Signaling Technology (Danvers, MA, US). Antibodies against MUC4, α-tubulin (GT114), Nanog (N3C3), and Sox2 (N1C3) were purchased from GeneTex (Hsinchu, Taiwan). Antibody against GAPDH was purchased from Proteintech (Rosemont, IL, US). Protein concentration was determined by the Bradford assay (Bio-Rad Laboratories, Hercules, Ca, US) before loading and verified by α-tubulin or GAPDH level at 1:100,000 dilution. The images were acquired by ChemiDoc MP Imaging System (Bio-Rad Laboratories, Hercules, Ca, US), and processed by Image Lab software (Version 5.2.1, Bio-Rad Laboratories, Hercules, Ca, US; https://www.bio-rad.com/en-us/product/image-lab-software?ID=KRE6P5E8Z).

### Surface CD44 staining

Cells were detached by Accutase (Sigma Aldrich, St. Louis, MI, US), and calculated in 1 × 10^6^ cells to be processed for CD44 staining. Cells were washed with chilled PBS twice and then incubated with blocking buffer (0.5% BSA, 2%FBS in PBS) for 30 min at room temperature. After wash with PBS twice, cells were incubated with anti-CD44 antibody (1:100 dilution) for 30 min at room temperature and protected from light. Cells were then washed with washing buffer (1% FBS and 1 mM EDTA in PBS) three times, and filtered with 40 µm mesh into tubes. The cells were ready for flow cytometry analysis (BD FACSCalibur). PE mouse anti-human CD44 (G44-26) and PE mouse IgG2b ĸ isotype (27–35) were purchased from BD Biosciences (San Jose, CA, US).

### Spheres formation assay

Cells were seeding at 1000 cells/well density in 96-well ultra-low attachment microplate (Corning, Corning, NY, US) in DMEM/F12 medium supplied with 1× B27 plus supplement, 1× N-2 supplement, 2 ng/ml bFGF, and 2 ng/ml EGF, all supplements were purchased from ThermoFisher (Waltham, MA, US). Cells was incubated at 37 °C for 14 days for estimation of sphere (> 100 nm) formation.

### MTT and MTS assay

Pancreatic cancer cells were seeded in a 96-well plate at 5000 cell density, incubated overnight. Cells were then treated with gemcitabine or other drugs for 48 h. Removed the medium and added 0.5 mg/ml MTT (Sigma Aldrich, St. Louis, MI, US) to each well and incubated for 3 h. Formazan was dissolved in DMSO and used for detection with absorbance at OD 570 nm. For MTS, 20 μl MTS (Abcam, Cambridge, UK) was added to wells and incubated for 3 h following gemcitabine treatment and detected by absorbance at OD 490 nm.

### Immunohistochemistry staining (IHC)

IHC assay was performed as previously described^11^. Antibodies used in IHC against IL-17RB (A81) were homemade. MUC1 (EP85) and MUC4 (EP256) were purchased from Bio SB (Santa Barbara**,** CA US). Results were grouped into low (< 10%) and high (> 10%).

### Statistical analysis

All data are presented as means ± SD, and Student’s *t* test was applied for comparison with the control group and other group. Significant statistic results are presented as *, **, and *** with *P* < 0.05, *P* < 0.01, and *P* < 0,001, respectively. Chi-squire test is performed by SPSS software (https://www.ibm.com/products/spss-statistics, version 18) and used for examining correlation among expression of IL-17RB, MUC1, MUC4, and clinical parameters in human subjects with pancreatic cancers.

## Supplementary information

Supplementary Information.

## Data Availability

All data to support the conclusions of this manuscript are included in the main text and supplementary materials. All materials are available upon request, including chemical compounds as supplies permit, and subject to a standard materials transfer agreement.

## Abbreviations

5-FU: 5-Fluorouracil
EMT: Epithelial–mesenchymal transition
IHC: Immunohistochemistry
IL17-RB: Interleukin-17 receptor B
MUC: Mucin
PanIN: Pancreatic intraepithelial neoplasia
PDAC: Pancreatic ductal adenocarcinoma
RT-qPCR: Quantitative real-time RT-PCR
