# Supplementary material for: Targeting interleukin-17 receptor B enhances gemcitabine sensitivity through downregulation of mucins in pancreatic cancer
Source: Sci Rep. 2020 Oct 20;10:17817. doi: 10.1038/s41598-020-73659-z (PMC7576602; doi:10.1038/s41598-020-73659-z)
Supplement: Supplementary file 1 — Supplementary Information. [file 41598_2020_73659_MOESM1_ESM.docx]

Supplementary information for

**Targeting Interleukin-17 Receptor B Enhances Gemcitabine Sensitivity through Downregulation of Mucins in Pancreatic Cancer**

Lung-Hung Tsai^1,2†^, Kai-Wen Hsu^1,2,3†^, Cheng-Ming Chiang^4^, Hsiu-Ju Yang^1^, Yu-Huei Liu^5^, Shun-Fa Yang^6^, Pei-Hua Peng^7^, Wei-Chung Cheng^1,2,8^, Heng-Hsiung Wu^1,2,8 *^

**Affiliations:**

^1^ Research Center for Cancer Biology, China Medical University, Taichung, Taiwan

^2^ Drug Development Center, China Medical University, Taichung, Taiwan

^3^ Institute of New Drug Development, China Medical University, Taichung, Taiwan

^4^ Simmons Comprehensive Cancer Center, Department of Pharmacology, and Department of Biochemistry, University of Texas Southwestern Medical Center, 5323 Harry Hines Boulevard, Dallas, Texas 75390, USA

^5^ Graduate Institute of Integrated Medicine, China Medical University, Taichung, Taiwan

^6^ Institute of Medicine, Chung Shan Medical University, Taichung, Taiwan

^7^ Cancer Genome Research Center, Chang Gung Memorial Hospital at Linkou, Taoyuan, Taiwan

^8^ Graduate Institute of Biomedical Sciences, China Medical University, Taichung, Taiwan

**^*^** Corresponding: Heng-Hsiung Wu, Ph.D. Email: [henghsiungwu@mail.cmu.edu.tw](mailto:henghsiungwu@mail.cmu.edu.tw)

^†^ Equal contribution

**Figure 1B**


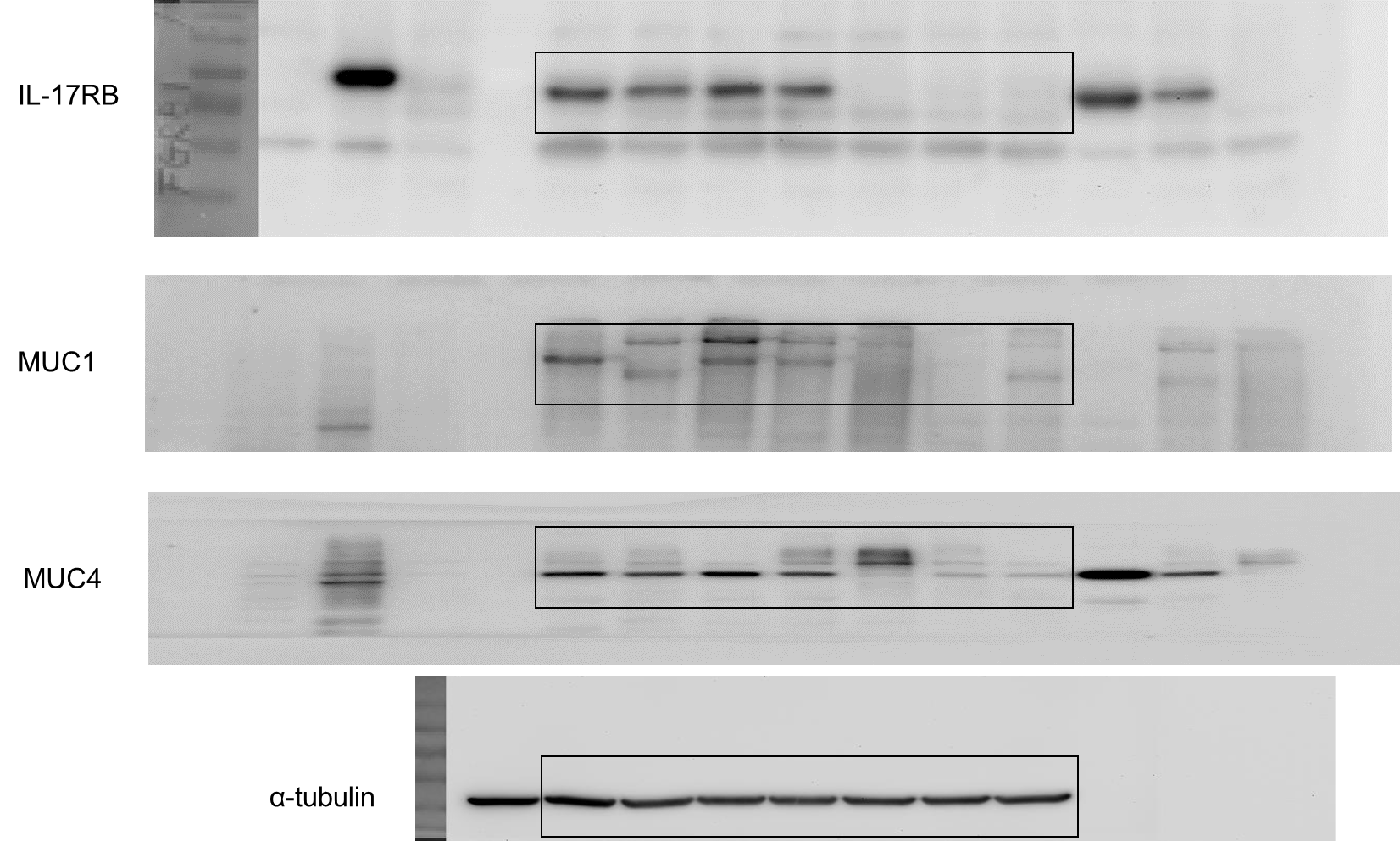


**Figure 1C**


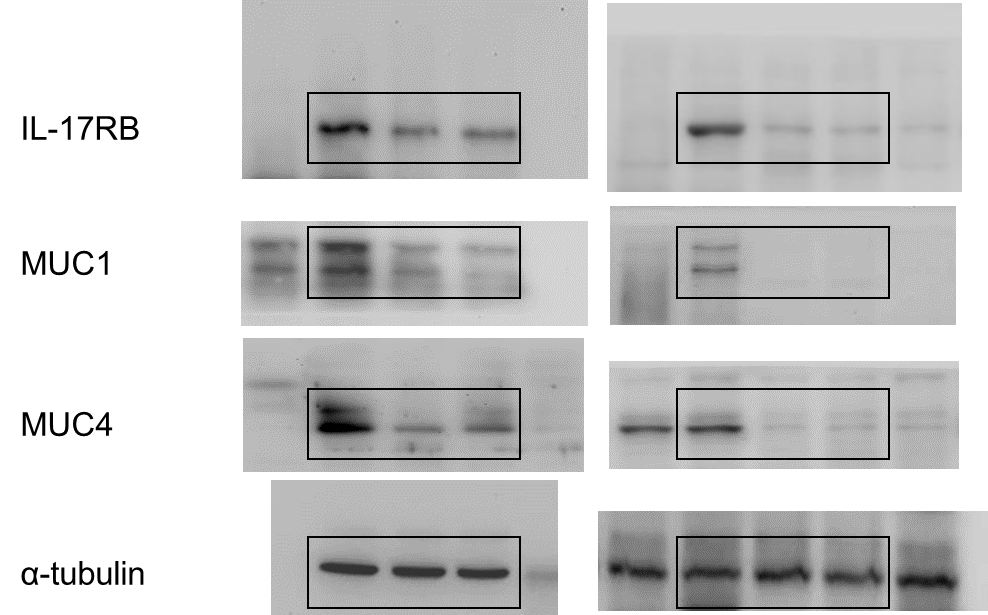


Supplementary Fig. 1. Full-length images of western blotting. Black rectangles
indicate regions used in the main figures.

**Figure 1F**


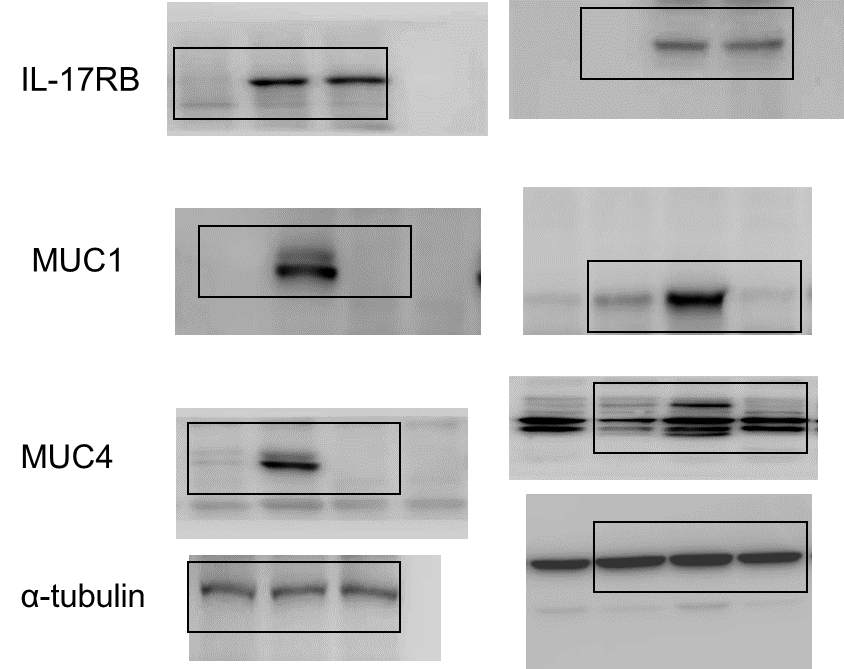


Supplementary Fig. 1 continued.

Figure 2A

**
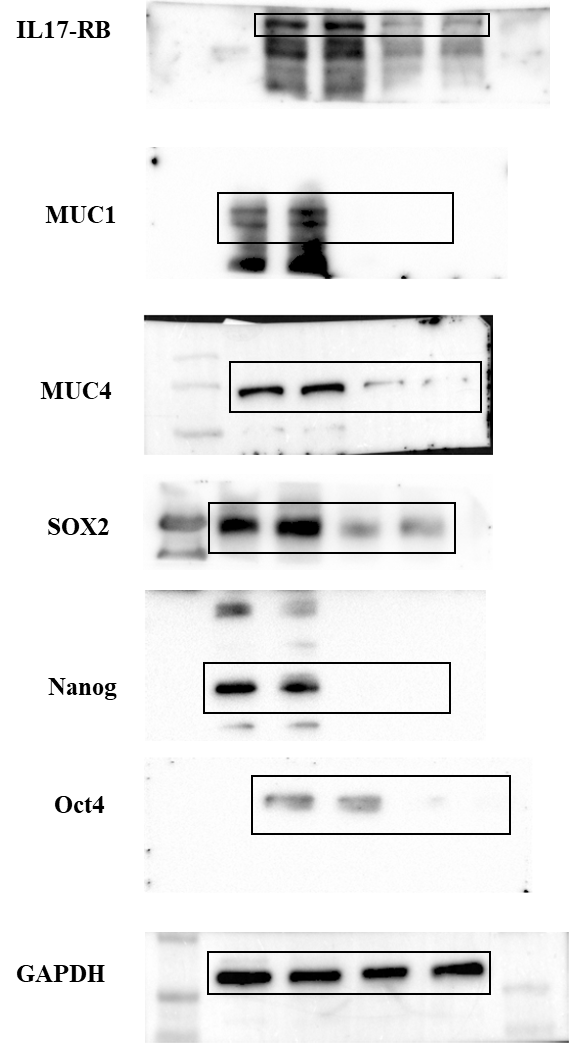
**

Supplementary Fig. 1 continued.

**Figure 2E**


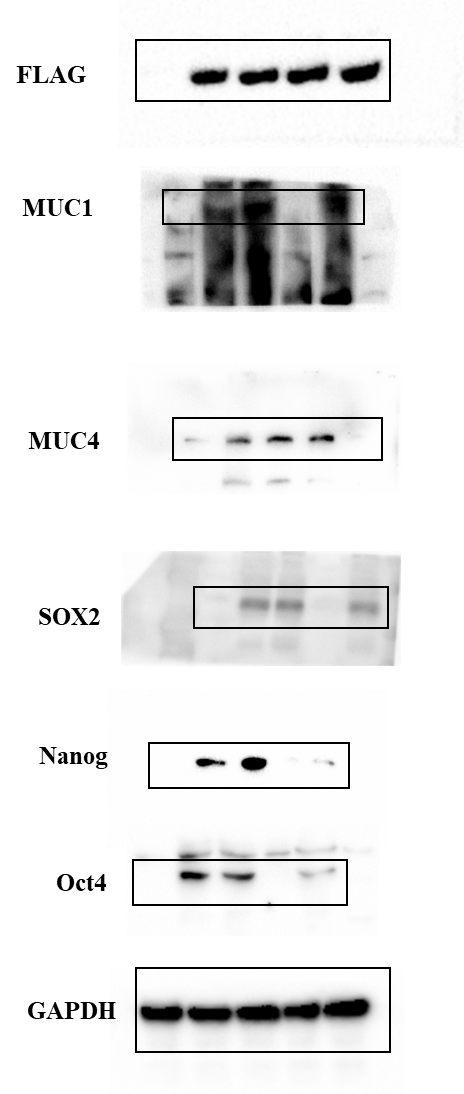


Supplementary Fig. 1 continued.

**Figure 4A**

**
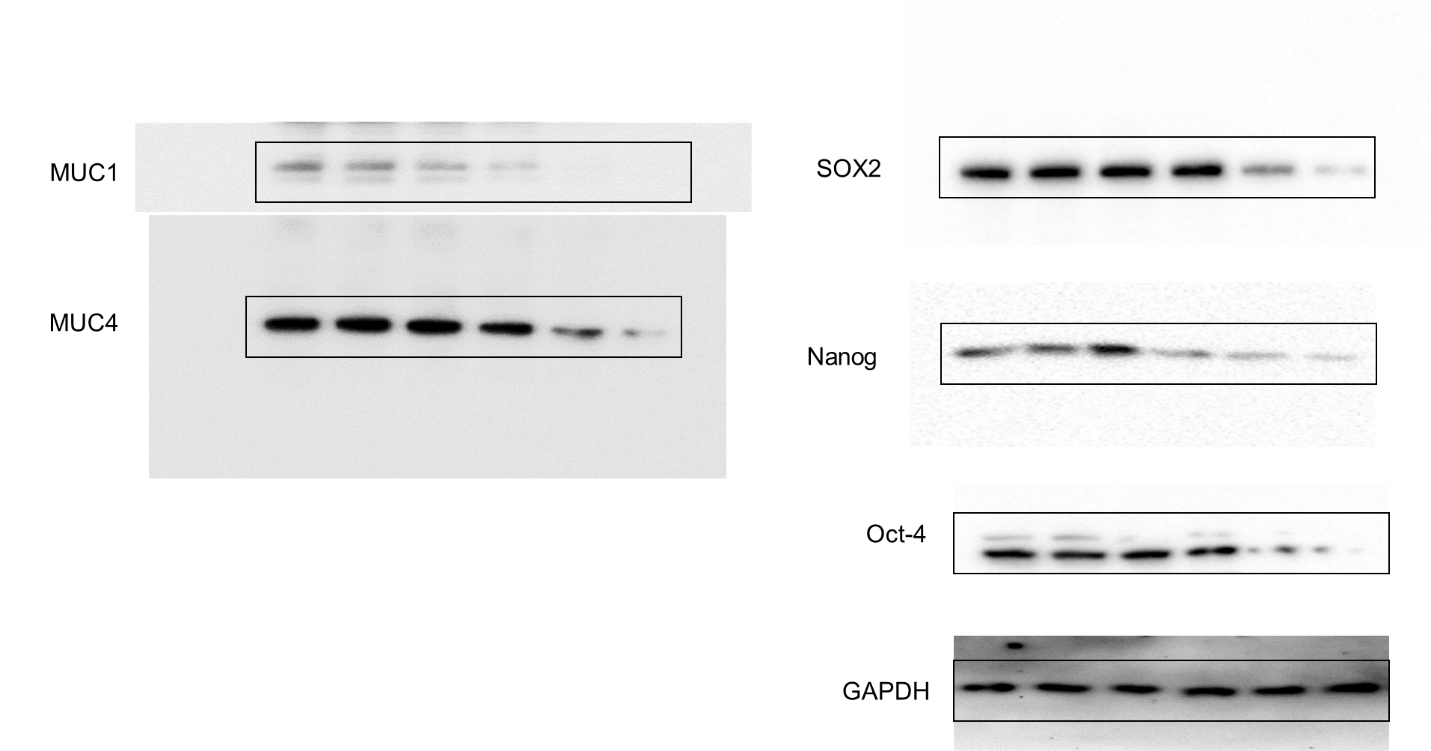
**

Supplementary Fig. 1 continued.

**

**

**Supplementary Fig. 2. mRNA level of MUC1 and MUC4 in pancreatic cell lines**

A panel of pancreatic cancer cell lines were harvested for RNA extraction, and synthesized to cDNA by using SuperScript IV reverse transcriptase kit (Thermo Fisher Scientific). The cDNA was used for MUC1 (A) and MUC4 (B) mRNA level analysis.


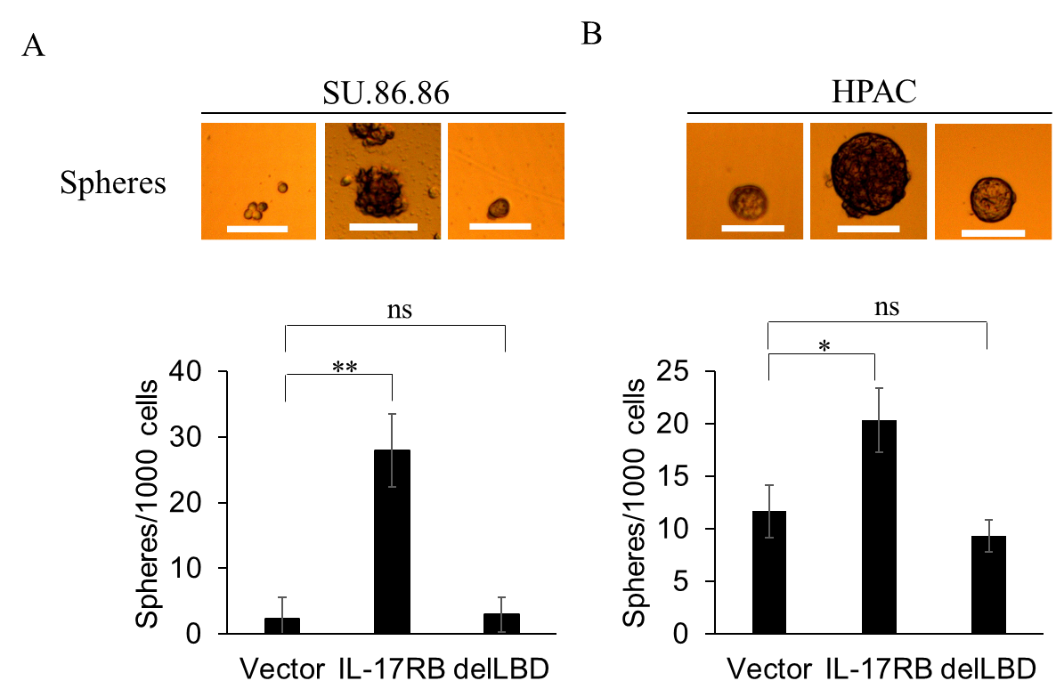


**Supplementary Fig. 3. Ligand-dependent of IL-17RB promotes sphere formation.**

Plasmids of vector control, wild-type IL-17RB, or ligand-binding domain deletion of IL-17RB were transfected into SU.86.86 and HPAC cells to measure sphere formation ability. Colony diameter more than 100 µm (scale bar) was counted as positive results. Asterisks indicate statistical significance (*, *P* < 0.05; **, *P* <0.01). ns, non-significant.


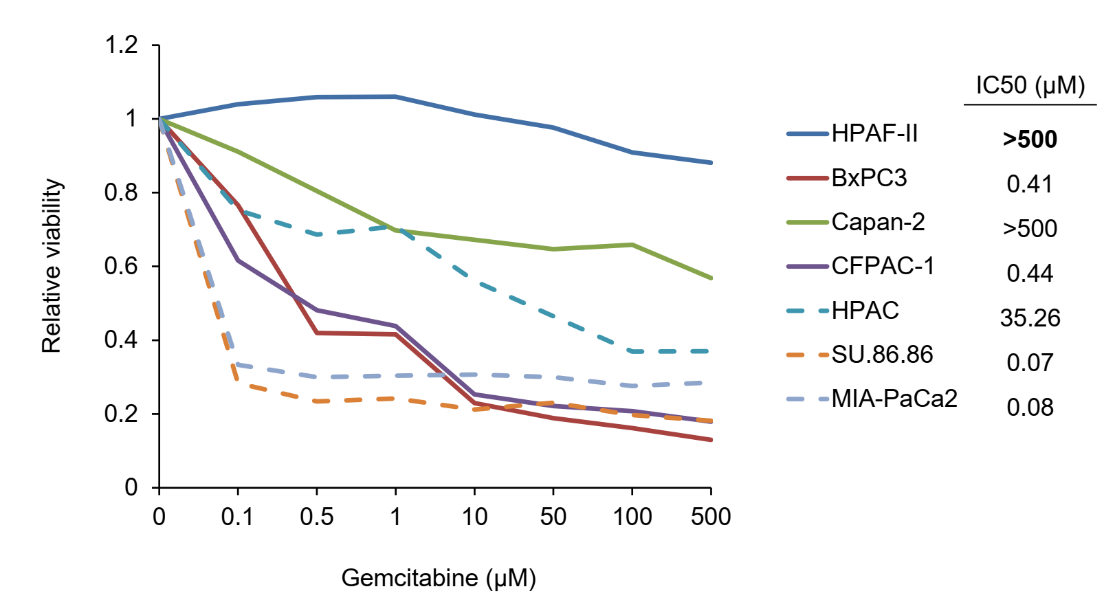


**Supplementary Fig. 4. IC50 of gemcitabine in pancreatic cancer cell lines**

Pancreatic cancer cell lines were treated with 0.1, 0.5, 1, 10, 50, 100, 500µM Gemcitabine for 48h to evaluate the 50% of inhibitory concentration (IC50). Cell viability was analyzed by MTT assay.





**Supplementary Fig. 5. Inhibition of NF-κB represses IL-17RB-regulated MUC1 and MUC4 mRNA expression**

BxPC3 (A and B) and IL-17RB-overexpressing SU.86.86 cells (C and D) were treated with 20 µM U0126, 20 µM PD98059, 10 µM BAY11-7082 for 48 h, RNA was extracted and analyzed for MUC1 and MUC4 mRNA expression by qPCR.

**Supplementary Table 1. Association between IL-17RB, MUC1, MUC4, and clinical parameters**

| Parameters | | N=91 | IL-17RB | |  | MUC1 | |  | MUC4 | |
| --- | --- | --- | --- | --- | --- | --- | --- | --- | --- | --- |
|  |  |  | High  n=44 | *P* value |  | High  n=36 | *P* value |  | High  n=17 | *P* value |
| Age | ≦52 | 46 | 22 (48 %) | 0.919 |  | 20 (44%) | 0.440 |  | 12 (26%) | 0.067 |
|  | >52 | 45 | 22 (49%) |  |  | 16 (36%) |  |  | 5 (11%) |  |
| Gender | Female | 35 | 16 (46%) | 0.691 |  | 11 (31%) | 0.210 |  | 4 (11%) | 0.161 |
|  | Male | 56 | 28 (50%) |  |  | 25 (45%) |  |  | 13 (23%) |  |
| Subtype | Duct Adeno. | 51 | 23 (45%) | 0.224 |  | 22 (43%) | 0.733 |  | 12 (24%) | 0.194 |
|  | Adeno. | 20 | 13 (65%) |  |  | 7 (35%) |  |  | 4 (20%) |  |
|  | Others | 20 | 8 (40%) |  |  | 7 (35%) |  |  | 1 (5%) |  |
| T | T1&T2 | 41 | 20 (49%) | 0.941 |  | 14 (34%) | 0.339 |  | 10 (24%) | 0.206 |
|  | T3&T4 | 50 | 24 (48%) |  |  | 22 (44%) |  |  | 7 (14%) |  |
| N | N0 | 83 | 35 (46%) | 0.114 |  | 32 (39%) | 0.527 |  | 15 (18%) | 0.631 |
|  | N1 | 8 | 6 (75%) |  |  | 4 (50%) |  |  | 2 (25%) |  |
| Stage | IA-IB | 38 | 18 (47%) | 0.874 |  | 12 (32%) | 0.187 |  | 9 (24%) | 0.300 |
|  | IIA-IV | 53 | 26 (49%) |  |  | 24 (45%) |  |  | 8 (15%) |  |
| Grade | 1 | 24 | 8 (33%) | 0.052 |  | 13 (54%) | 0.773 |  | 6 (25%) | 0.964 |
|  | 2 | 23 | 12 (52%) |  |  | 11 (48%) |  |  | 5 (22%) |  |
|  | 3 | 25 | 17 (68%) |  |  | 11 (44%) |  |  | 6 (24%) |  |

*P* value was calculated by Chi-squire analysis.
